# Perinatal Exposure to Environmental Tobacco Smoke (ETS) Enhances Susceptibility to Viral and Secondary Bacterial Infections 

**DOI:** 10.3390/ijerph9113954

**Published:** 2012-10-31

**Authors:** Jocelyn A. Claude, Amy Grimm, Hannah P. Savage, Kent E. Pinkerton

**Affiliations:** 1 Center for Health and for the Environment, University of California, Davis, One Shields Avenue, Davis, CA 95616, USA; Email: jaclaude@ucdavis.edu; 2 School of Veterinary Medicine, University of California, Davis, One Shields Avenue, Davis, CA 95616, USA; Email: agrimm@ucdavis.edu (A.G.); hpsavage@ucdavis.edu (H.S.); 3 Department of Pediatrics, University of California, Davis, One Shields Avenue, Davis, CA 95616, USA

**Keywords:** environmental tobacco smoke (ETS), perinatal, influenza A, *S. aureus*

## Abstract

Studies suggest childhood exposure to environmental tobacco smoke (ETS) leads to increased incidence of infections of the lower respiratory tract. The objective of this study was to determine whether perinatal exposure to ETS increases the incidence, morbidity and severity of respiratory influenza infection and whether a secondary bacterial challenge at the peak of a pre-existing viral infection creates an enhanced host-pathogen susceptibility to an opportunistic infection. Timed-pregnant female Balb/c mice were exposed to either ETS for 6 h/day, 7 d/week beginning on gestation day 14 and continuing with the neonates to 6 weeks of age. Control animals were exposed to filtered air (FA). At the end of exposure, mice were intranasally inoculated with a murine-adapted influenza A. One week later, an intranasal inoculation of *S. aureus* bacteria was administered. The respective treatment groups were: bacteria only, virus only or virus+bacteria for both FA and ETS-exposed animals for a total of six treatment groups. Animal behavior and body weights were documented daily following infection. Mice were necropsied 1-day post-bacterial infection. Bronchoalveolar lavage fluid (BALF) cell analysis demonstrated perinatal exposure to ETS, compared to FA, leads to delayed but enhanced clinical symptoms and enhanced total cell influx into the lungs associated with viral infection followed by bacterial challenge. Viral infection significantly increases the number of neutrophils entering the lungs following bacterial challenge with either FA or ETS exposure, while the influx of lymphocytes and monocytes is significantly enhanced only by perinatal ETS exposure. There is a significant increase in peribronchiolar inflammation following viral infection in pups exposed to ETS compared with pups exposed to FA, but no change is noted in the degree of lung injury between FA and ETS-exposed animals following bacterial challenge. The data suggests perinatal exposure to ETS alters the response of neonates to the timing and severity of infection as well as ETS alters the pattern of inflammation and cellular influx into the lungs due to viral and bacterial infection.

## 1. Introduction

Secondhand smoke, also known as environmental tobacco smoke (ETS), remains a major public health concern. In 2006, the US Surgeon General’s report concluded that there is no safe level of exposure to tobacco smoke. Smoking has been banned in many public places with the positive result that many people’s exposure to ETS has dramatically declined. However children remain susceptible to the highest levels of ETS exposure because it is legal to smoke in one’s home and automobile [1,2,3]. Children are highly susceptible to infections early in life due to immature immune, metabolic and respiratory systems. ETS exposure during this time is associated with adverse health effects, including increases in the incidence of middle ear infections, asthma, and lower respiratory infections. In addition, secondary bacterial infections during or following viral infection are a common cause of virus related death in infants [4]. In mice, influenza virus infections predispose mice to secondary bacterial pneumonias [5]. 

**Figure 1:**
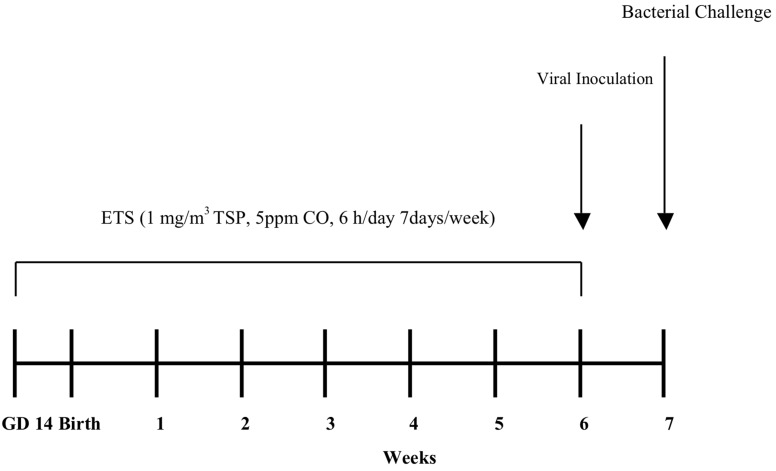
Experimental timeline. Balb/c moms began ETS exposure on gestation day 14. After birth on gestation day 21, moms and pups continued exposure. By three weeks of age, pups were weaned from their mother, while ETS exposure continued to 6 weeks of age followed by an intranasal viral infection (week 6) and an intranasal bacterial infection 1 week later (week 7). Mice were sacrificed 1-day post bacterial infection. Control animals were exposed to filtered air.

The mechanism by which ETS exposure during the perinatal period enhances susceptibility of infants and young children to respiratory infection is unknown. The objective of this study is to determine whether perinatal exposure to ETS increases the incidence, morbidity, and severity of respiratory influenza infection as well as whether a secondary bacterial challenge at the peak of a pre-existing viral infection creates an enhanced host-pathogen susceptibility to an opportunisitic infection. Pregnant mice were exposed to ETS during gestation for 1 week before birth, and their pups were subsequently exposed to ETS for 6 weeks post-natally. At 7 weeks of age, pups were given an influenza viral inoculation followed by a *Staphylococcus aureus* challenge 1 week later. Animals were sacrificed 24 h post-bacterial infection. A pictorial illustration of the experimental timeline is presented in Figure 1. We found that perinatal exposure to ETS alters the timing and severity of infection as well as the pattern of inflammation and cellular influx in the lungs due to viral and bacterial infection. 

## 2. Materials and Methods

### 2.1. Animals

Thirty-four timed-pregnant female Balb/c mice were purchased from Harlan Laboratories at gestation day 14. Animals were housed one female per cage at the Center for Health and the Environment at the University of California, Davis. Sixty female pups were used to conduct this study assigned randomly to 6 groups of 10 pups each. 

### 2.2. Tobacco Smoke Exposure

Sixteen timed-pregnant mice and their pups were exposed only to filtered air (FA) only for 24 h 7d/week for the duration of the study. Eighteen timed-pregnant mice were exposed daily to tobacco smoke for 6 h/day. After birth, these dams and pups continued exposure to ETS together until weaning. The pups continued ETS exposure until 6 weeks of age. Research cigarettes (3R4F, University of Kentucky) were burned at a rate of two cigarettes every 10 min with a puff volume of 35 mL over 2 s, once per minute. Both sidestream and mainstream cigarette smoke were collected via a chminey and passed to a dilution and aging chamber to achieve the target concentration of ETS (1.0 ± 0.17 mg/m^3^). The carbon monoxide level was 4.8 ± 0.8 ppm, and the average temperature was 73 °F. For this study, 60 female mice offspring (30 from ETS exposed mothers and 30 from FA exposed mothers) were randomly assigned to three different treatment groups (bacteria-only, virus-only, or virus followed by bacteria) per inhaltion exposure (ETS or FA) for a total of six experimental groups. 

### 2.3. Influenza Virus Inoculation

A mouse adapted viral strain of A/PR/8/34 (H1N1) influenza was obtained as a generous gift from Melinda Beck (University of North Carolina, Chapel Hill, NC, USA). The stock solution was diluted to a dose of 50 TCID_50_ (tissue culture infective dose) in 40 µL of PBS for intranasal inoculation. Mice in the bacteria-only infection groups received 40 µL of PBS. Behavior and body weight were observed and recorded for 7 days following inoculation. The 7 day endpoint was chosen because during the course of a viral infection the adaptive immune response is highest at this time point, as mature lymphocytes are introduced into the circulation. A clinical score based on behaviorial observations was used to assess morbidity [6,7,8]. One point was scored for each sign of illness. Signs scored were unkempt fur, lethargy, hunched posture, and shivering. At the end of the 7 day observation period, the points given to each animal were tallied on a per day basis. These totals were used to determine the group daily means. 

### 2.4. Bacteria Inoculation

*Staphylococcus aureus* ATCC strain 29,253 (methicillin-susceptible) was obtained from American Type Culutre Collection (ATCC, Manassas, VA, USA) and cultured for 24 h at 37 °C on sheep blood agar plates. Seven days after inoculation with either influenza virus or PBS, mice were intranasally inoculated with 40 µL of *S. aureus*, at a concentration of 10^8^ CFU per 100 µL saline. Mice in the influenza-only infection groups received 40 µL of saline. Mice were necropsied 24 h post-*S. aureus* infection. 

### 2.5. Bronchoalveolar Lavage (BAL)

Lungs (n = 10/group) were lavaged three times with Hanks buffered salt solution (HBSS). The first aliquot was 0.6 mL followed by two aliquots of 0.5 mL. The recovered bronchoalveolar lavage fluid (BALF) was centrifuged for 15 min at 2,000 RPM and resuspended in 500 µL of Hanks. Total cell counts were performed using a hemocytometer. Cell differential counts were done on cytospin preparations using Diff-Quik stain. 

### 2.6. Lung Histology and Scoring

Following BAL, lungs (n = 5/group) were inflated with 4% paraformaldehyde at 30 cm of pressure for 1 h using a tracheal cannula. Right lung lobes were separted, embedded in paraffin, sectioned 5 µm thick, and stained with hematoxylin and eosin (H&E) on slides. Since viral infection alone involves both the airways and the parenchyma, while bacterial infection is mainly found in the airways, histological evaluation included the airways, blood vessels, alveoli, pleura and associated structures for each lung lobe. Lung tissues from mice exposed to ETS + virus and FA + virus were graded for the extent and severity of inflammation, both in the alveoli and in the bronchiolar submucosa, and a total score of severity mutiplied by extent was calculated for alveolar and bronchiolar inflammation per lobe. Extent of the alveolar inflammation was estimated for each lung lobe as “none”, “<25%”, “25–50%”, or” >50%”, corresponding to grades of 0–3, respectively. The affected and total number of bronchioles in each lung lobe was counted. The percentage of affected bronchioles was used to estimate the extent of bronchiolar submucosal inflammation. Severity for both alveolar and bronchiolar inflammation per lobe was estimated to be “none”, “a few inflammatory cells”, “a moderate number of inflammatory cells”, or “complete consolidation”, corresponding to grades of 0–3, respectively. If severity of the inflammtion varied, the extent was estimated for each region of inflammation and the scores were added. The scoring of all the slides was done by an evaluator without knowledge of the treatment group. 

### 2.7. Statistical Analysis

Data are expressed as the mean ± standard error of the mean (SEM). Analysis of variance (ANOVA) and Tukey honest significant difference (Tukey HSD) tests were used to determine statistically significant differences between groups using using JMP 9.0 software (SAS Institute, Inc., Cary, NC, USA). The Tukey honest significance test compares the means of each group to the means of all the other groups. A *p*-value < 0.05 was considered statistically significant. For this study, a sample size of 10 for each treatment group was used, which allowed for a sufficient confidence in findings of enhanced susceptibility to viral and bacterial infection following an exposure condition to ETS in mice. This to gives us a power calculation which determines statistically significant changes at the 97% confidence level.

## 3. Results and Discussion

**Figure 2:**
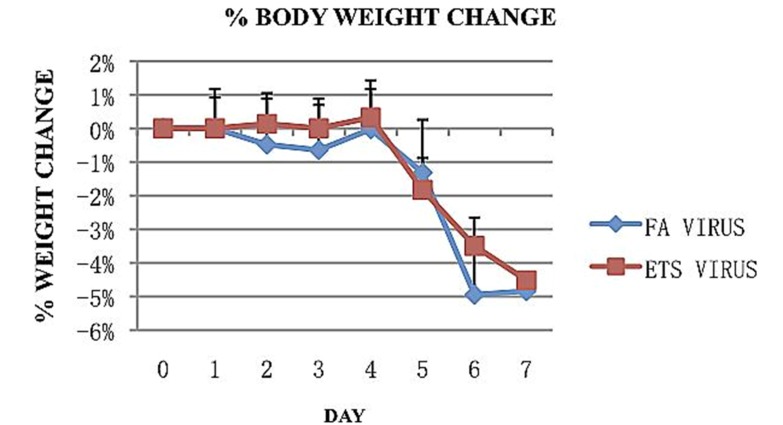
Percent change in body weight following inoculation with influenza A virus. Both exposure groups showed a similar decrease in total percent weight in the 7 days following viral inoculation. Both groups reached a total decrease of about 5% by day 7.

Weight and symptoms of illness were observed and recorded daily during the 7 days following viral inoculation. Animal weight remained fairly stable for the first 4 days, with less than a 1% change in the total percent body weight. Thereafter, it began a quick decline for both FA and ETS exposed animals (Figure 2).

**Figure 3:**
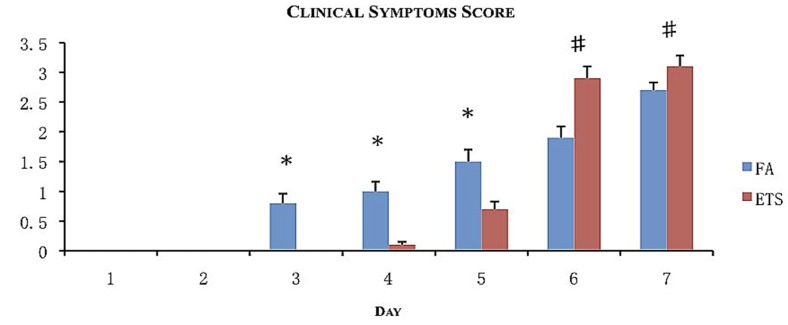
Morbidity assessment following viral inoculation with exposure to filtered air (FA) or environmental tobacco smoke (ETS). Clinical scores were calculated by assigning one point for every sign of illness observed for 7 days following viral inoculation. Data is presented as the group mean (n = 20) ±SEM. * indicates a significantly increased degree of clinical scoring compared to ETS day-matched exposure groups (*p* < 0.05). # indicates a significantly increased degree of clinical scoring compared to FA day-matched exposure groups (*p* < 0.05).

All animals showed signs of illness. Symptoms included unkempt fur, lethargy, shivering, and hunched posture. For every symptom observed, a point was given to calculate the clinical score in order to assess the morbidity (Figure 3). The most common symptom was unkempt fur. This was also the first symptom to appear follwed by lethargy and hunched posture. Only animals exposed to ETS demonstrated shivering behavior. 

Onset of the symptoms varied according to exposure (FA or ETS). FA exposed groups began to show symptoms 3 days post-inoculation, whereas ETS exposed animals began to show symptoms 5 days post-inoculation.

Symptoms gradually increased for FA animals, while ETS animals experienced larger daily increases. Therefore, despite the increased time for symptoms to appear, ETS animals had achieved a clinical score that was statistically higher than FA animals by days 6 and 7 (Figure 3). This observation suggests for animals exposed to ETS experience a more severe infection (through combined clinical symptoms) than animals exposed to FA. 

These results suggest that ETS exposure may cause a delay in immune function leading to later onset of symptoms and signs of infection. A study by Noakes *et al*. found that neonatal exposure to ETS alters the functioning of the neonate innate immune system. Tobacco smoke exposure affects cytokine expression and signaling receptors, which can affect the type of response mounted by an individual to specific types of pathogenic invasions [9]. This time delay may be one reason many young children are hospitalized due to viral infection; by the time parents realize a child is sick there is no time to take preventative measures and the virus has already to began to run its course. 

**Figure 4:**
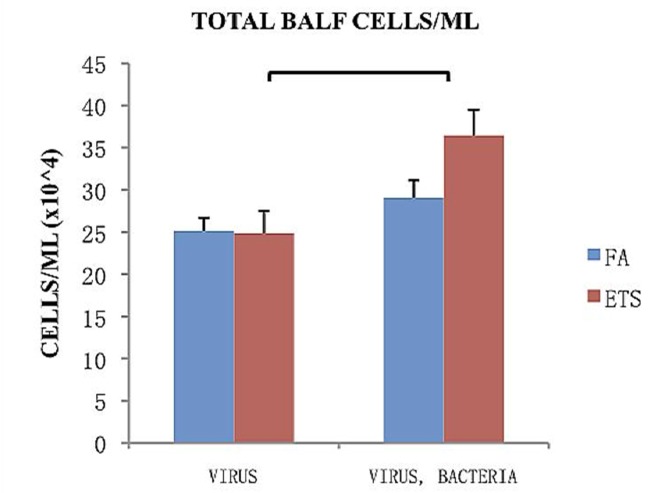
Total cells/mL collected from BALF 24-h post-bacterial infection. Virus groups had the same amount of cells 8 days following viral inoculation, while groups treated with both virus and bacteria showed an increase in the number of cells 8 days following viral inoculation and 1 day post bacterial infection. Data is presented as the group mean (n=10) ±SEM. The bracket between bars designates a statistically significant difference between the ETS-virus and ETS-virus-bacteria groups (*p* < 0.05).

The total number of cells collected from the bronchoalveolar lavage fluid (BALF) of animals treated with virus only or with an additional challenge to *S. aureus* is shown in Figure 4. Within each infection group (virus only or virus + bacteria) no significant difference was found between FA and ETS. However, there was a highly significant increase in the total number of cells associated with secondary bacterial infection in mice exposed to ETS that was not observed in the FA group (Figure 4). This finding strongly suggests that ETS exposure during early life increases susceptibility to secondary bacterial infection. 

**Figure 5:**
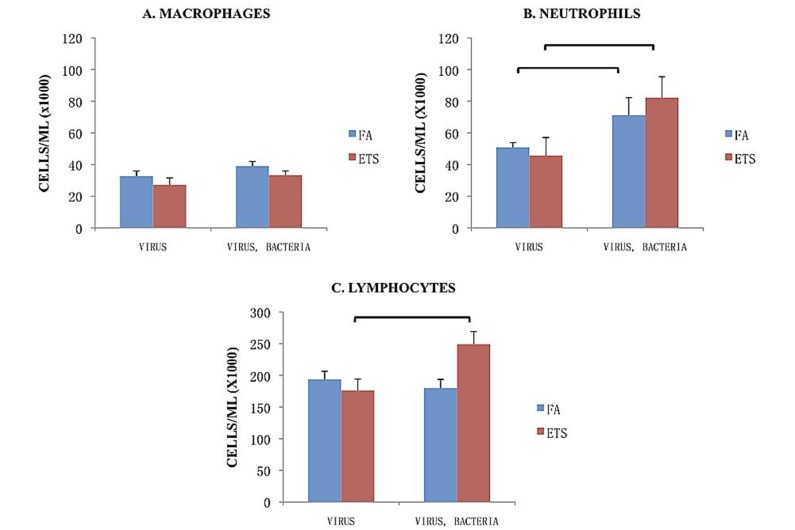
Breakdown of cell types in the BALF. The numbers presented in these graphs represent the cell number/mL found in BALF. Data is presented as the group mean (n=10) ±SEM. The bracket between designated bars signifies a statistically significant difference between the FA-virus and FA-virus-bacteria and ETS-virus and ETS-virus-bacteria groups (*p* < 0.05).

The division of leukocyte counts (macrophages, neutrophils, lymphocytes) within the BALF is shown in Figure 5. Neither inhalation exposure or pathogen exposure caused differences in macrophage recruitment between the groups (Figure 5(A)). Neutrophil recruitment was significantly greater in mice subsequently exposed to bacteria following viral infection for both mice exposed to FA or to ETS (Figure 5(B)). 

Lymphocyte/monocyte recruitment accounted for the largest portion of cells recovered in BALF (Figure 5(C)). Compared to FA exposure, perinatal ETS exposure significantly enhanced the number of lymphocytes and/or monocytes recovered by BAL following viral + bacterial infection. This finding suggests an enhanced humoral response to the infection, as well as stimulating a significant influx of lymphocytes and/or monocytes into the lungs. 

**Figure 6:**
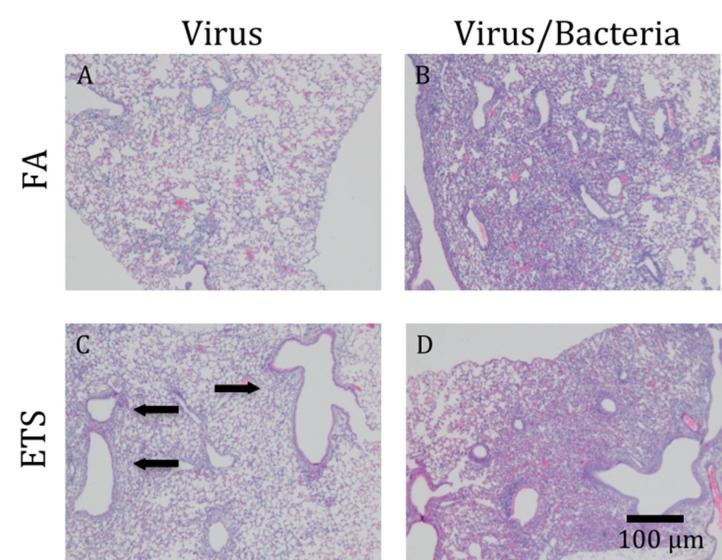
Lung pathology. Hemotoxylin and eosin staining was done on fixed right lung tissue. All lobes were stained. Images presented here photographed at 5×. (**A**) Virus+FA causes patches of mild inflammation, mostly found in the alveoli. (**C**) Virus+ETS caused increased inflammation around the bronchioles (arrows), as compared to virus+FA. (**B**) and (**D**) show that a secondary bacterial infection causes a greater influx of cells to become localized around the airways and throughout the parenchyma.

To determine the effect that bacterial infection alone could have on leukocyte recruitment, 10 female neonatal mice were given a *S. aureus* infection and sacrificed at 1 day post-infection. The mice were found to develop a marked inflammatory response accompanied by a moderate, patchy degree of edema and consolidation with the presence of macrophages and abundant neutrophils (data not shown). This pattern of inflammation was slightly reduced from that observed in mice first infected with virus, followed by a secondary bacterial challenge (Figure 6).

H&E staining was performed on fixed right lung tissue to determine histological changes following perinatal exposure to FA *versus* ETS (Figure 6). Due to the overwhelming effect of acute bacterial innoculation/infection, there were no significant histological differences seen between the lungs of mice exposed to FA, virus + bacteria compared with ETS, virus+bacteria (Figure 6(B) *vs*. Figure 6(D)). The lungs from all bacterially-infected animals showed great celluler (*i.e.*, leukocyte) recruitment, which involved about 15% of parenchyma. No signs of tissue damage were seen. 

**Figure 7:**
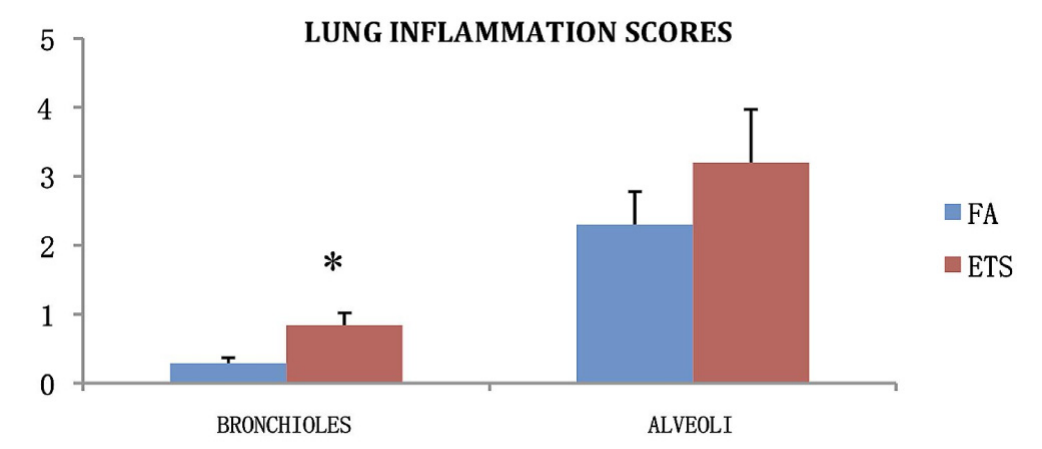
Average inflammation scores for each lung lobe of virus only infected animals. Scores are calculated as extent times severity of inflammation, for both the alveoli and the bronchiolar sub mucosa. Data is presented as the group mean (n = 10) ±SEM. * indicates a significant difference between the FA and ETS groups in that category.

Inflammation scores were calculated for peribronchiolar inflammation in animals exposed to virus only to determine the inflammation effects of the virus. Animals exposed to ETS had a significantly greater inflammation score for bronchioles compared to animals exposed to FA (Figure 7). 

## 4. Conclusions

The objective of this study was to determine whether perinatal exposure to ETS increases the incidence, morbidity and severity of respiratory influenza infection, as well as whether a secondary bacterial challenge at the peak of a pre-existing viral infection creates an enhanced host-pathogen susceptibility to an opportunistic infection. Previous research has shown that influenza virus predisposes mice to secondary bacterial pneumonias [5]. We found that perinatal exposure to ETS not only enhanced secondary bacterial infection (*S. aureus*) in the presence of a murine-adapted influenza A virus, but also altered the pattern of influenza illness. While neonatal animals exposed to ETS had a delayed onset of signs of influenza infection compared to neonatal animals exposed to FA, the onset of these signs was significantly more severe. 

The exposure conditions used in our study in mice are within a relevant particle concentration range that children might experience in homes where smoking occurs. Actual exposure conditions of children to ETS in the home or other settings can be highly variable. Our study demonstrates clear biological plausibility for enhanced susceptibility to viral and bacterial infection in children who might be exposed under similar conditions. Still, it may be difficult to extrapolate the conditions of exposure to ETS used in these mice to predict the precise degree of infectivity in children. 

Perinatal ETS exposure was associated with an overall increase in inflammatory cells recruited following the secondary bacterial infection, a slightly elevated neutrophil response, and a highly significant increase in lymphocyte and/or monocyte recrutiment to the lungs. These findings suggest that ETS exposure may have caused a delay in immune function leading to later onset of viral symptoms and signs of infection. Noakes *et al*. found that neonatal exposure to ETS alters the neonatal innate immune system by affecting cytokine expression and signaling receptors, which can influence the type of response mounted against specific types of pathogens [9,10,11]. The altered immune response timing and increased severity of infection seen in mice in our study could also be the result of some of the observed effects of pre- and postnatal human exposure to ETS, such as decreased lung growth, thickening of the airways, epithelial lining damage and changes to cytokines produced in the fetoplacental unit [12,13,14]. All of these changes can potentially increase a child’s susceptibility to infection, resulting in a later, but more aggressive onset of symptoms, which may account for the increased hospitalization of young children compared to other age groups. These observations are clearly evident in our study in neonatal mice with more severe clinical symptoms during the later stages of viral infection that may enhance the actions of subsequent infection due to an opportunistic bacterial pathogen, which we found to lead to elevated pulmonary inflammation and numbers of immunoeffector cells entering the lungs as detected in increased BAL values. 
